# Cannabinoid-Induced Inhibition of Morphine Glucuronidation and the Potential for In Vivo Drug–Drug Interactions

**DOI:** 10.3390/pharmaceutics16030418

**Published:** 2024-03-18

**Authors:** Shelby Coates, Keti Bardhi, Philip Lazarus

**Affiliations:** Department of Pharmaceutical Sciences, College of Pharmacy and Pharmaceutical Sciences, Washington State University, 412 E. Spokane Falls Blvd, Spokane, WA 99202, USA

**Keywords:** cannabinoids, morphine, drug–drug interactions, AUCR, cannabis, UGT, opioids, THC, CBD, IVIVE

## Abstract

Opioids are commonly prescribed for the treatment of chronic pain. Approximately 50% of adults who are prescribed opioids for pain co-use cannabis with their opioid treatment. Morphine is primarily metabolized by UDP-glucuronosyltransferase (UGT) 2B7 to an inactive metabolite, morphine-3-glucuronide (M3G), and an active metabolite, morphine-6-glucuronide (M6G). Previous studies have shown that major cannabis constituents including Δ^9^-tetrahydrocannabinol (THC) and cannabidiol (CBD) inhibit major UGT enzymes. To examine whether cannabinoids or their major metabolites inhibit morphine glucuronidation by UGT2B7, in vitro assays and mechanistic static modeling were performed with these cannabinoids and their major metabolites including 11-hydroxy-Δ^9^-tetrahydrocannabinol (11-OH-THC), 11-nor-9-carboxy-Δ^9^-tetrahydrocannabinol (11-COOH-THC), 7-hydroxy-cannabidiol (7-OH-CBD), and 7-carboxy-cannabidiol (7-COOH-CBD). In vitro assays with rUGT-overexpressing microsomes and human liver microsomes showed that THC and CBD and their metabolites inhibited UGT2B7-mediated morphine metabolism, with CBD and THC exhibiting the most potent K_i,u_ values (0.16 µM and 0.37 µM, respectively). Only 7-COOH-CBD exhibited no inhibitory activity against UGT2B7-mediated morphine metabolism. Static mechanistic modeling predicted an in vivo drug–drug interaction between morphine and THC after inhaled cannabis, and between THC, CBD, and 7-OH-CBD after oral consumption of cannabis. These data suggest that the co-use of these agents may lead to adverse drug events in humans.

## 1. Introduction

**Figure 1:**
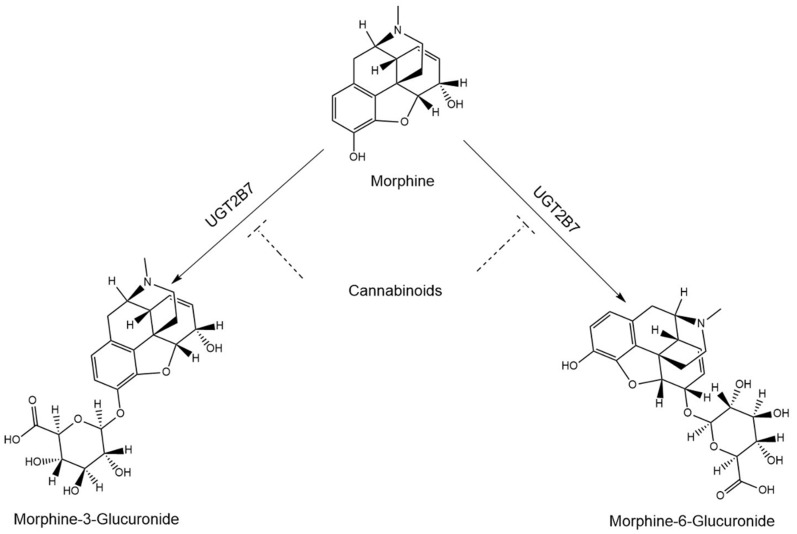
Hypothesized inhibition of morphine metabolism by cannabinoids.

Morphine is a common opioid analgesic used to treat chronic pain and high-impact chronic pain [1,2], and is used in a variety of other medical settings (e.g., cancer) [3]. Morphine is primarily metabolized by UDP-glucuronosyltransferase (UGT) 2B7 to form its major metabolite, morphine-3-glucuronide (M3G), and an active metabolite, morphine-6-glucuronide (M6G; see Figure 1) [3,4,5,6], which is thought to contribute to the analgesic efficacy of morphine as it has a greater affinity for mu-opioid receptors than its parent drug [7,8]. Morphine is also metabolized to a small extent by cytochrome P450 (CYP) 3A and sulfatases to the inactive metabolites, normorphine [9], and morphine-3- and morphine-6-sulfate, respectively [10].

Cannabis is federally classified as an illicit substance in the United States, but it has been legalized in 38 states, the District of Columbia, and three territories for medicinal use; in 24 states, two territories, and the District of Columbia, cannabis has been legalized for recreational use [11]. Recent studies have shown that cannabis has grown in popularity to treat a number of different medical conditions, with retrospective studies showing that almost 40% of patients surveyed use cannabis to treat unspecified chronic pain [12]. Studies show that individuals who use opioids for chronic pain often report using cannabis in combination with opioids [13,14]. Previous research has found that ~50% of adults who are prescribed opioids for their pain management, use cannabis regularly with opioids [14,15]. Due to the increasing use of cannabis, especially in the chronic pain population, determining the potential for pharmacokinetic drug–drug interaction (DDI) between morphine and cannabis is crucial for patient safety.

Cannabis contains numerous phytochemicals, termed cannabinoids. The main psychoactive cannabinoid, Δ^9^-tetrahydrocannabinol (THC), elicits the ‘high’ that many people use cannabis for [16,17,18]. Cannabidiol (CBD) and cannabinol (CBN) are also major cannabinoids that have been shown to have various pharmacological effects, including antiepileptic effects, anti-inflammatory effects, and pain relief [16,19,20,21,22,23]. Once inside the body, these cannabinoids are metabolized to various phase I and phase II metabolites. THC is metabolized to an active metabolite, 11-hydroxy-Δ^9^-tetrahydrocannabinol (11-OH-THC), that is also psychoactive [24]. 11-OH-THC is further metabolized to 11-nor-9-carboxy-Δ^9^-tetrahydrocannabinol (11-COOH-THC) [24,25], which can then undergo glucuronidation to 11-nor-Δ^9^-tetrahydrocannabinol-carboxylic acid glucuronide (THC-COO-Gluc) [26]. Similarly, CBD is metabolized to an active metabolite, 7-hydroxy-cannabidiol (7-OH-CBD) [27,28], which is then further metabolized to 7-carboxy-cannabidiol (7-COOH-CBD) [28,29]. Similar to that observed for 11-COOH-THC, 7-COOH-CBD undergoes glucuronidation to form 7-carboxy-cannabidiol glucuronide (7-COO-Gluc). While high THC and CBD plasma levels are observed within 30–60 min after cannabinoid exposure, clinical studies have shown that their metabolites including 11-OH-THC, 11-COOH-THC, 7-OH-CBD, and 7-COOH-CBD, also reach high levels [28,29,30,31]. The plasma concentrations and areas under the curve (AUC) of these metabolites exceed those of both parent compounds (THC and CBD) by ≥1.25 fold and in the case of the carboxy metabolites ≥2.5 fold [28,31].

Including phase II metabolic enzymes in DDI studies is becoming increasingly important. UGTs account for 40–70% of clinical drug metabolism [32,33]. Although DDI studies focus primarily on phase I metabolism, examining DDIs involving UGTs becomes increasingly important if a drug relies primarily on UGT-mediated metabolism [34,35]. Impaired clearance of a drug through glucuronidation can cause undesired effects, including build-up of parent drug, resulting in various toxicities. For example, a DDI has been observed via UGT2B7-mediated inhibition after coadministration of zidovudine (AZT) and methadone, resulting in the inhibition of zidovudine glucuronidation and subsequent increases in zidovudine exposure by 1.4-fold [36].

Previous studies using probe substrates have shown that cannabinoids like THC and CBD as well as THC metabolites like 11-OH-THC are potent inhibitors of numerous CYP enzymes [37,38,39,40,41,42,43,44,45]. In similar studies, THC and CBD were also shown to be potent inhibitors of UGT enzymes, specifically UGTs 1A9, 1A6, 2B4, and 2B7 [38]. The goal of the present study was to examine the potential DDI between UGT-mediated morphine metabolism and major cannabinoids and cannabinoid metabolites and the potential clinical significance of these inhibitory effects.

## 2. Materials and Methods

### 2.1. Chemicals and Materials

THC, 11-OH-THC, 11-COOH-THC, CBD, 7-OH-CBD, 7-COOH-CBD, and CBN were purchased and obtained from Cayman Chemicals (Ann Arbor, MI, USA) or Sigma-Aldrich (St. Louis, MO, USA) after obtaining approval from the Drug Enforcement Administration. Morphine, morphine-3-glucuronide, morphine-6-glucuronide, morphine-3-glucuronide-D_3_, and morphine-6-glucuronide-D_3_ were purchased from Sigma-Aldrich (St. Louis, MO, USA). Pooled human liver microsomes (HLMs; 50 subjects, mixed sex) were purchased from Sekisui Xenotech, LLC (Lenexa, KS, USA). Ultra-low-binding microcentrifuge tubes as well as high-performance-liquid-chromatography-grade ammonium formate, methanol, and formic acid were purchased from Thermo Fisher Scientific (Waltham, MA, USA). Ketoconazole was purchased from Sigma-Aldrich (St. Louis, MO, USA) while uridine diphosphate glucuronic acid (UDPGA) was purchased from Cayman Chemical (Ann Arbor, MI, USA). Bovine serum albumin (BSA) was purchased from Sigma-Aldrich (St. Louis, MO, USA). Dulbecco’s Modified Eagles Medium (DMEM), Dulbecco’s phosphate-buffered saline (DPBS), and geneticin (G418) were purchased from Gibco (Grand Island, NY, USA), while premium-grade fetal bovine serum (FBS) was purchased from Seradigm (Radnor, PA, USA). The ACQUITY UPLC HSS T3 (1.8 µM × 2.1 mm × 100 mm) column used for ultra-pressure liquid chromatography–mass spectrometry (UPLC-MS) was purchased from Waters (Milford, MA, USA).

All in vitro and mathematical modeling experiments were performed as follows.

### 2.2. Inhibition Screenings of Cannabinoids and Their Metabolites as Inhibitors of UGT2B7

The UGT2B7 (H268)-overexpressing human embryonic kidney (HEK) 293 cell line has been previously developed and described [46] and was verified by Sanger sequencing. The parent HEK293 cell line used in this study was purchased from ATCC in 2015 and authenticated by ATCC in 2017 using short-tandem repeat polymorphism analysis. *Mycoplasma* was not detected in these cells in 2021. Microsomal membrane fractions were prepared from HEK293 cells overexpressing recombinant UGT2B7 by differential centrifugation, as previously described [46,47]. Total microsomal protein concentrations were determined using the BCA assay per the manufacturer’s instructions. UGT2B7 inhibition assays (final volume = 50 µL) were performed in reactions contained 20–100 µg of total microsomal protein, 25 mmol/L Tris buffer, 2 mmol/L MgCl_2_, 2% BSA, and 275 µM morphine (i.e., the known K_M_ for UGT2B7 against morphine) [48]. An individual cannabinoid or cannabinoid metabolite was added as a potential inhibitor at two different concentrations, 10 and 100 µM. Microsomes were preincubated with alamethicin (50 µg/mg of microsomal protein) on ice for 15 min prior to incubation. Reactions were initiated by the addition of 4 mM UDPGA and incubated for 1–1.5 h at 37 °C. Each experiment had a positive control reaction containing 10 or 100 µM of probe inhibitor (ketoconazole), which was added instead of cannabinoid. The relative activity of a given reaction was measured against a reaction containing only vehicle (3% methanol) and no inhibitor.

To reduce the non-specific binding of cannabinoids to labware, low-bind microcentrifuge tubes were used for all reactions. Additionally, 2% BSA was added to sequester inhibitory long-chain unsaturated fatty acids that are known to inhibit the activity of UGT2B7 [49,50] and increase cannabinoid solubility [24,51]. Reactions were terminated with the addition of 50 µL of ice-cold methanol containing internal standards (morphine-3-glucuronide-D_3_ and morphine-6-glucuronide-D_3_). Samples were then centrifuged for 30 min at 17,000× *g* at 4 °C. Supernatants were then collected and run on the UPLC-MS/MS, as described below.

Inhibition assay conditions were optimized for overexpressing cell lines and HLM (see below) for both reaction time and microsomal protein added. Optimal conditions were based on the following criteria: (1) metabolite formation was linear with enzyme concentration and time, (2) substrate depletion was < 20% during the incubation, and (3) metabolite formation detection with the UPLC-MS/MS method was reproducible.

### 2.3. IC_50_ Determinations

For the cannabinoids and metabolites that exhibited ≥50% inhibition of either morphine-3-glucuronide or morphine-6-glucuronide formation at either the 10 µM or 100 µM cannabinoid concentrations, *IC*_50_ values were determined in UGT2B7-overexpressing HEK293 cell microsomes and in HLM. The assay parameters were the same as those described above for the inhibition screenings, with a range of 10–12 cannabinoid inhibitor concentrations used between 0.1 and 250 µM. *IC*_50_ values were determined from three independent experiments.

Cannabinoids and their metabolites are highly lipophilic and have extensive non-specific binding to labware and microsomal protein [52]. To account for the non-specific binding of cannabinoids to both labware and protein, fraction unbound determinations previously reported by our lab [37,38] were used in the present studies; the fraction unbound calculations for 7-OH-CBD and 11-COOH-THC were based off of those observed previously for 11-OH-THC. *IC*_50_ values were corrected for non-specific binding by cannabinoids ${(IC}_{50,u})\;$ by calculating the unbound fraction in the incubation for individual cannabinoids (*f_u,inc_*) in either HLM or HEK293 microsomes (Equation (1)).

(1) $${IC}_{50,u}={IC}_{50}\times f_{u,inc}$$

*K_i_* values were corrected for non-specific binding by cannabinoids in a similar fashion as the *IC*_50_ values, as shown in Equation (2).

(2) $$K_{i,u}=K_{i}\times f_{u,inc}$$

### 2.4. UPLC-MS/MS Analysis

UPLC-MS/MS was performed with mobile phases consisting of 5 mmol/L ammonium formate in water with 0.1% UPLC-MS/MS-grade formic acid (buffer A) and methanol with 0.1% UPLC-MS/MS-grade formic acid (buffer B). A UPLC-HSS T3 (1.8 µM × 2.1 mm × 100 mm) column with a flow rate of 0.400 mL/min was used to separate morphine-3-glucuronide and morphine-6-glucuronide as follows: 30 s at 100% A, 30 s at 95% A, 4 min at 25% A, 30 s at 5% A, and re-equilibration for 1.5 min at 100% A. The injection volume was 1–5 µL with a column temperature of 30 °C. MS/MS detection was performed in a Waters ACQUITY XEVO TQD instrument in MRM ESI+ mode. The MS/MS scans were performed using the following mass transitions: morphine-3-glucuronide (*m*/*z* 462.2000 > 286.2000), morphine-6-glucuronide (*m*/*z* 462.2000 > 286.3000), morphine-3-glucuronide-D_3_ (*m*/*z* 465.2000 > 289.2000), and morphine-6-glucuronide-D_3_ (*m*/*z* 465.2000 > 289.5000), respectively. The collision energy was optimized to 36 V for both morphine-3-glucuronide and morphine-6-glucuronide. A cone voltage of 30 V and 0.025 s dwell time resulted in the high-sensitivity detection of both morphine glucuronide metabolites. The desolvation temperature was 500 °C, with 800 L/hour of nitrogen gas. Glucuronide metabolite retention times observed in the enzymatic incubations were compared to the deuterated glucuronide metabolite internal standards retention times. All metabolite concentrations were quantified using Targetlynx software (version 4.1, Waters Acquity) by interpolation from matrix-matched standard curves (0.0036 ppm–7.5 ppm) prepared using standards and deuterated internal standards. The accuracy of all standard curves was R^2^ = 0.998.

### 2.5. Static Mechanistic In Vitro to In Vivo Extrapolation IVIVE

Assuming the worst-case scenario (maximum inhibition among total possible mechanisms of inhibition), a competitive-type inhibition scenario was used to determine the *K_i_* value (inhibition constant) [53]. *K_i_* values were generated using Equation (3), as the inhibition assay incubations were performed at the apparent *K_m_* for UGT2B7:

(3) $$K_{i}=\frac{{IC}_{50}}{2}$$

The DDI potential from inhibition of drug metabolizing enzymes was assessed using static mechanistic modeling as recommended by the FDA [53], which assesses the potential for a DDI in vivo by comparing the area under the concentration time curve (*AUC*) in the presence and absence of inhibitor to generate the AUC ratio (*AUCR*; Equation (4)):

(4) $$AUCR=\left(\frac{1}{A_{g}\times \left(1-F_{g}\right)+F_{g}}\right)\times \left(\frac{1}{A_{h}\times f_{m}+\left(1-f_{m}\right)}\right)$$

where *F_g_* is the fraction available after intestinal metabolism and was set to 1 as recommended by the FDA [53] and *f_m_* is the specific enzyme contribution to metabolism of morphine to its respective metabolites and set to 0.7 (total morphine glucuronidation) [54]. As recommended by the FDA, an *AUCR* cutoff ≥ 1.25 was used to indicate the potential for a DDI in vivo [53].

(5) $$A_{g}=\frac{1}{1+\frac{{\left[I\right]}_{g}}{K_{i,u}}}$$

(6) $${\left[I\right]}_{g}=\frac{F_{a}\times K_{a}\times Dose}{Q_{en}}$$

Equation (5) defines *A_g_* as the effect of reversible inhibitions in the intestine on the substrate drug (morphine). *[I]_g_* is the inhibitor concentration in the intestine and is defined in Equation (6), where *F_a_* is the fraction absorbed after oral administration, which was set to 1 as recommended by the FDA [53] for inhalation and 1 for THC and CBD after oral administration, respectively. *K_a_* is the first-order absorption rate constant in vivo and was set to 0.02 [45].

Equation (7) defines *A_h_* as the effect of reversible inhibitions in the liver on the substrate drug (morphine):

(7) $$A_{h}=\frac{1}{1+\frac{{\left[I\right]}_{h}}{K_{i,u}}}$$

where *[I]_h_* is the inhibitor concentration in the liver and is defined in Equation (8), with *f_u,p_* the unbound fraction of inhibitor in plasma, and *C_max_* is the maximal total (free and bound) inhibitor concentration in the plasma.

(8) $${\left[I\right]}_{h}=f_{u,p}\times \left(C_{max}+\frac{F_{a\;}\times F_{g}\times K_{a}\times Dose}{Q_{h\;}\times R_{b}}\right)$$

For the static mechanistic modeling, hepatic blood flow (*Q_h_*) and enterocyte blood flow (*Q_en_*) were set to 1500 mL/min and 300 mL/min, respectively, and the blood-to-plasma concentration ratio (*R_B_*) was set to 0.4 [55]. A range of THC and CBD doses [THC: 20–160 mg; CBD: 19–2000 mg [56]] were used to simulate average low and high doses of THC or CBD through both oral and inhalation routes of administration (Supplemental Table S1).

A *f_m_* value equivalent to the total glucuronidation of morphine by UGT2B7 was used to calculate the AUCR. The *K_i_* calculated previously for morphine-3-glucuronide formation was used in the static mechanistic modeling since morphine-3-glucuronide is the predominant morphine glucuronide isomer formed in plasma of subjects administered morphine [57].

### 2.6. Data Analysis

Data were exported and analyzed using Excel (Microsoft Version 2402). The amount of metabolite formation at each concentration of cannabinoid relative to the no-inhibitor control (i.e., percent metabolite formation) was calculated using the ratio of peak area of sample/ peak area of internal standard to obtain the peak area of metabolite with and without inhibitor ratios. Ratios were subsequently normalized to the percent of the control (no inhibitor), which were calculated using the peak area of metabolite with inhibitor ratio/peak area of metabolite without inhibitor ratio × 100%. The metabolite peak area was normalized to respective internal standard peak area.

IC_50_ values were calculated by plotting the percentage of metabolite formation (metabolite peak area with inhibitor normalized to the respective internal standard peak area divided by the normalized metabolite peak area without inhibitor) versus the log concentration of each cannabinoid inhibitor, evaluated using GraphPad Prism 7.04 software (GraphPad Software Inc., San Diego, CA, USA). IC_50_ curves were constrained to 100 and 0 for the top and bottom of the curves, respectively. K_i_ values were calculated for each IC_50_ value (determined in triplicate) such that each independently determined IC_50_ value was used to calculate independent K_i_ values so that a standard deviation could be generated.

## 3. Results

**Figure 2:**
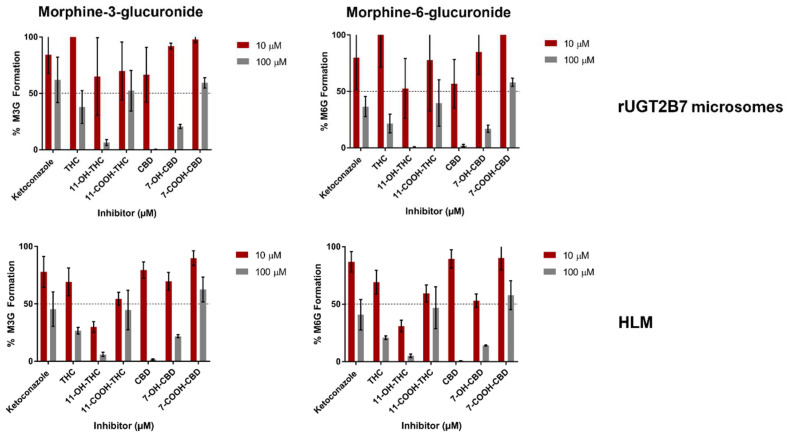
Inhibition assay screening of morphine-3-glucuronide and morphine-6-glucuronide formation in rUGT2B7 microsomes (**upper panels**) and HLM (**lower panels**).

Inhibition screenings of THC, 11-OH-THC, 11-COOH-THC, CBD, 7-OH-CBD, 7-COOH-CBD, and CBN as potential inhibitors of morphine metabolism in microsomes from rUGT2B7-overexpressing cells showed that at 100 µM, THC inhibited M3G and M6G formation by 62% and 78%, respectively (Figure 2). Similar levels of inhibition were observed with 100 µM 11-COOH-THC (48% and 60% inhibition against M3G and M6G formation, respectively). Greater inhibition was observed with 11-OH-THC, CBD, and 7-OH-CBD, with 100 µM CBD decreasing M3G and M6G formation by 99% and 98%, respectively, 11-OH-THC decreasing M3G and M6G formation by 93% and 99%, respectively, and 7-OH-CBD decreasing M3G and M6G formation by 80% and 84%, respectively. Weak inhibition of M3G and M6G formation was observed for 100 µM 7-COOH-CBD (40% and 42% inhibition, respectively); CBN did not result in appreciable inhibition of either M3G or M6G formation in rUGT2B7 microsomes, exhibiting decreases of 10% and 33% glucuronidation activity, respectively.

Inhibition screening results in rUGT2B7-overexpressing microsomes were confirmed in inhibition screening assays performed with commercially available pooled HLM (Figure 2). Similar to that observed in UGT2B7-overexpressing microsomes, 100 µM 11-COOH-THC and 7-COOH-CBD exhibited weak to moderate inhibition of M3G and M6G formation. THC, 11-OH-THC, CBD, and 7-OH-CBD all exhibited significant inhibition of both M3G and M6G formation in HLM at 100 µM. Like that observed for UGT2B7-overexpressing microsomes, CBN did not exhibit appreciable inhibition of M3G and M6G formation in HLM.

The concentration-dependent inhibition of morphine metabolism by THC, 11-OH-THC, 11-COOH-THC, CBD, and 7-OH-CBD was determined in both rUGT2B7-overexpressing microsomes and HLM by establishing *IC_50_* values for each cannabinoid that exhibited at least 50% inhibition at 100 µM in the inhibition screening assays. Due to the high lipophilicity of cannabinoids [52], *IC*_50_ values were corrected for nonspecific binding of the individual cannabinoids tested in the experimental system. The unbound fraction (*f_u,inc_*) values of THC, 11-OH-THC, and CBD in overexpressing HEK293 microsomes were determined in previous studies, with a range in *f_u,inc_* of 0.038 (CBD) to 0.078 (11-OH-THC) for rUGT-overexpressing microsomes and 0.048 (THC) to 0.094 (11-OH-THC) for HLM [38]. Due to similarities in structure, the *f_u,inc_* for 11-OH-THC was used as a surrogate for both 11-COOH-THC and 7-OH-CBD [38].

**Figure 3:**
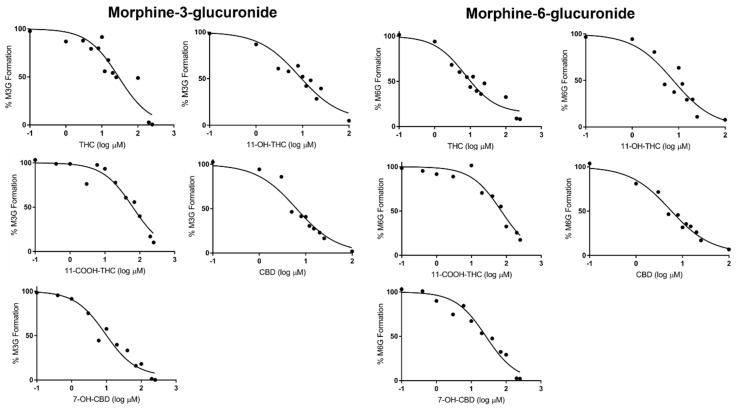
Representative *IC_50_* curves in rUGT2B7 microsomes against morphine-3-glucuronide and morphine-6-glucuronide formation.

Representative IC_50_ curves are shown for each cannabinoid in rUGT2B7-overexpressing microsomes in Figure 3 and HLM in Supplemental Figure S1. CBD exhibited the most potent inhibition of both M3G and M6G metabolite formation in UGT2B7-overexpressing microsomes, with unbound cannabinoid fraction-corrected *IC_50_* values (IC_50,u_) of 0.35 ± 0.17 µM and 0.19 ± 0.06 µM, respectively (Table 1); similar *IC_50,u_* values were observed in HLM (0.42 ± 0.23 µM and 0.32 ± 0.13 µM for M3G and M6G formation, respectively). THC and 11-OH-THC also exhibited potent inhibition of both M3G and M6G formation in both UGT2B7-overexpressing microsomes and HLM. 7-OH-CBD and 11-COOH-THC exhibited more moderate inhibition of both M3G and M6G metabolite formation in UGT2B7 microsomes, with IC_50,u_ values 10- and 12-fold higher in UGT2B7-overexpressing microsomes and HLM, respectively, as compared to CBD. Interestingly, while the *IC_50,u_* values for 11-COOH-THC were higher in HLM than that observed in rUGT2B7-overxpressing microsomes, the *IC_50,u_* values were 3- to 7-fold lower in HLM for 7-OH-CBD. In addition, while the *IC_50,u_* values were similar for M3G and M6G formation for all cannabinoids in rUGT2B7-overexpressing microsomes, they were consistently lower for M6G formation as compared to M3G formation in HLM.

**Figure 4:**
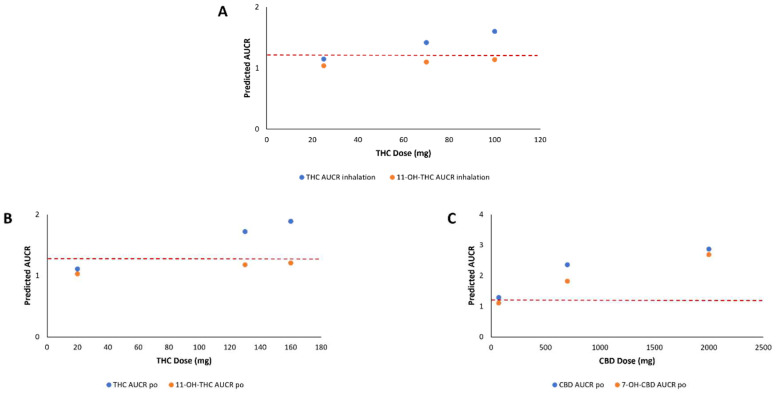
Drug interaction risks predicted utilizing static mechanistic models associated with co-use of morphine and increasing clinical doses of THC and CBD. (**A**), THC and 11-OH-THC after inhalation of THC; (**B**), THC and 11-OH-THC after oral (po) administration of THC; (**C**), CBD and 7-OH-CBD after oral (po) administration of CBD. Dashed lines indicate the AUCR cutoff of ≥ 1.25 as recommended by the FDA.

Mechanistic static modeling populated with calculated K_i,u_ values (Table 2) from the present inhibition studies were used to predict in vivo DDI between THC, CBD, and their metabolites when THC or CBD is co-administered with morphine by comparing the exposure of morphine in the presence of cannabinoids or their metabolites and the exposure of morphine alone. After inhalation of 70 mg of THC or oral administration of 130 mg of THC, a DDI was predicted to occur. Following the oral administration of CBD at a prescribed dose of 1000 mg twice daily (2000 mg total), a DDI was predicted for both CBD and 7-OH-CBD. No DDI was predicted with the THC metabolites 11-OH-THC and 11-COOH-THC, or with CBD, after inhaled dosing of THC or CBD, respectively, and no DDI was predicted after oral consumption of THC at low, medium, and high doses for 11-OH-THC and 11-COOH-THC (Table 3). The THC/morphine AUCRs for inhaled or oral administered THC, as well as the CBD/morphine and 7-OH-CBD/morphine AUCRs after oral administered CBD, increased proportionally with an increase in cannabinoid dose when co-administered with morphine and reached the AUCR ≥ 1.25 FDA cutoff to indicate a DDI in vivo (Figure 4). A marginal increase in AUCR was observed for 11-OH-THC with an increase in THC dose but did not reach an AUCR ≥ 1.25.

## 4. Discussion

The present study is the first comprehensive investigation of the inhibitory effects of major cannabinoids and their metabolites against the UGT2B7-mediated metabolism of morphine. Results from this study indicate that the major cannabinoids THC and CBD as well as several of their metabolites (11-OH-THC and 7-OH-CBD) exhibit strong inhibition of the UGT2B7-mediated metabolism of morphine. The THC metabolite 11-COOH-THC exhibited more moderate inhibition of UGT2B7, while no significant inhibitory activity was observed for CBN. Although morphine is also metabolized by cytochrome P450 (CYP) 3A, sulfotransferases (SULTs), and other UGTS (1A1, 1A3, and 1A8) [4,6,9,10,58,59], the enzyme with the largest *f_m_* is UGT2B7 (>70%) [54]. Thus, we focused on examining the potential inhibition of morphine metabolism with the enzyme that would result in the greatest clinical impact (i.e., UGT2B7) if it were to be inhibited.

Interestingly, significant inhibition of UGT2B7 was not observed in previous studies using the UGT2B7-specific probe substrate, AZT, by 11-COOH-THC, or 7-OH-CBD [38]. This difference suggests substrate specificity may be important in the inhibition of UGT2B7 activity by these cannabinoids. Furthermore, of the cannabinoids that showed inhibition of UGT2B7, the *IC_50,u_* values determined in Nasrin et al. (2021) [38] using probe substrates significantly varied from those determined using morphine, which is also considered a probe substrate for UGT2B7 [50,60]. The difference between the previously reported values with AZT is between 2- and 9-fold higher than those reported in the present study with morphine. This suggests that relying solely on DDI studies performed with probe substrates is not sufficient to characterize a potential DDI for all known UGT2B7 substrates. Therefore, further studies are needed to characterize the potential DDIs between cannabis and other known UGT2B7 substrates to better characterize the inhibitory effect cannabinoids have on UGT2B7-mediated metabolism. The level of inhibitory effect that cannabinoids may have on UGT2B7 substrates will likely vary due to the promiscuity of UGT2B7, as it can metabolize a wide range of structurally diverse compounds that contain an alcohol for *O*–glucuronidation [61].

Of the cannabinoids that exhibited inhibition, THC and CBD exhibited the strongest inhibition of both M3G and M6G formation in rUGT2B7-overexpressing microsomes. Inhibition potency was similar for M3G and M6G inhibition for the cannabinoids tested, with CBD > THC > 7-OH-THC > 11-OH-THC > 11-COOH-THC. Interestingly, inhibition potency of both 11-OH-THC and 7-OH-CBD were the same against both M3G and M6G, unlike the other cannabinoids where inhibition of M6G formation was most potent. In HLM, CBD again exhibited the strongest inhibition of both M3G and M6G formation. The potency of each cannabinoid against both metabolites was similar in both HLM- and rUGT2B7-overexpressing microsomes, with the exception of 11-COOH-THC which, unlike that observed for rUGT2B7-overexpressing microsomes, favored inhibition of M6G formation over M3G formation. The cannabinoid potency against both M3G and M6G formation in HLM was as follows: CBD > THC > 7-OH-CBD > 11-OH-THC > 11-COOH-THC. This was similar to the cannabinoid potency seen in rUGT2B7-overexpressing microsomes.

For the majority of cannabinoids tested, the *IC_50,u_* values in rUGT2B7-overexpressing microsomes and HLM were similar, indicating a good correlation between HLM and the recombinant system utilized in the present studies. Of the cannabinoids that exhibited similar *IC_50,u_* values in both systems, the *IC_50,u_* values in rUGT2B7-overexpressing microsomes were generally lower and more potent than those observed for HLM, suggesting that cannabinoids may be more potent inhibitors of UGT2B7 activity against morphine than other UGTs present in HLM that may exhibit some morphine glucuronidation activity. For 7-OH-CBD, the *IC_50,u_* values in rUGT2B7-overexpressing microsomes were greater and less potent than those determined in HLM, suggesting that this cannabinoid metabolite may be a less potent inhibitor against UGT2B7 as compared to other morphine glucuronidating UGTs. Interestingly, *IC_50,u_* values against M6G formation were consistently more potent as compared to *IC_50,u_* values against M3G formation. This may be due to the rate of metabolite formation where M3G is formed five times faster than M6G [57]. This finding suggests potential stereoselectivity in the inhibitory effects of the cannabinoids where this trend followed. This includes THC, 11-OH-THC, CBD, and 7-OH-CBD.

The *K_i,u_* values observed for the majority of the cannabinoids and their metabolites evaluated in the present study against both M3G and M6G formation approach the physiologically relevant concentration range of cannabinoids after consumption [THC *C_max,u_* = 0.02 µM [62]; CBD *C_max,u_* = 0.13 µM [31]. This suggests that unwanted DDI may occur in individuals who co-use morphine and cannabis (and/or CBD) via the inhibition of morphine-mediated UGT2B7 metabolism. The exception is for 11-COOH-THC, where *K_i,u_* values are significantly higher than known physiological levels after consumption. However, the 11-COOH-THC *K_i,u_* values were still lower than described in previous studies, showing no significant inhibition of AZT glucuronidation in both UGT2B7-overexpressing microsomes and HLM [38]. Together, these data are consistent with the data observed from mechanistic static modeling using THC or CBD doses from previous clinical trials, which demonstrated that THC, CBD, and 7-OH-CBD showed the potential for a DDI in vivo with morphine. The predicted increase in morphine exposure with coadministration of THC or CBD was > 1.25-fold but was < 3-fold (Table 3). Although this would be classified as a “weak” DDI [53], morphine has a narrow therapeutic index [3] and a 2-fold increase in morphine exposure (as predicted with coadministration with CBD) will likely necessitate a reduction in morphine dosage to avoid adverse side effects. However, there are limitations to the mechanistic static modeling approach used in the present study. This includes that there are few published clinical trials studying the pharmacokinetics of major cannabinoids and their metabolites. As current studies utilize similar doses (20 mg to 100 mg of THC) in single-dose studies [63], the doses chosen for mechanistic static modeling in the present study may not accurately depict the pharmacokinetics of major cannabinoids and their metabolites in cannabis users who are more frequent or chronic users. Studies have shown that there are usage differences between occasional and chronic users (dosage and frequency) along with individual differences in inhalation depth, breath hold, etc., lead to pharmacokinetic and disposition differences [64]. Differences in cannabinoid pharmacokinetics and disposition due to these factors may make overall clinical predictions of potential DDI with cannabinoids difficult. Another factor to consider is the cannabis products on the market vary in cannabinoid content, potency, and route of administration, further complicating clinical predictions. Clinicians should monitor patient populations that are known to frequently co-use cannabis with their medications (i.e., chronic pain and anxiety) [12]. Furthermore, the equation used as recommended by the FDA (Equation (2)) is limited in its function, as only one inhibitor can be investigated at a time with a victim drug. In contrast, when cannabis is consumed, many cannabinoids including THC, CBD, and many of their metabolites will all be present in plasma. Future studies will be required to examine the inhibitory effects on drug-metabolizing enzymes with the combined use of cannabinoids and metabolites in dynamic models to best predict the clinical impact of this potential DDI. Physiologically based pharmacokinetic (PBPK) modeling can be implemented to improve predictions by including multiple inhibitors in the DDI model (i.e., THC/CBD and its metabolites). PBPK models allow for the pharmacokinetics of the inhibitors (unlike static modeling that assumes the worst case scenario by incorporating the *C_max_* of the perpetrator drug and the inhibitor concentration is assumed to be constant) to be taken into consideration to give a dynamic concentration of the inhibitor(s) over time and, thus, gives a more realistic prediction of the potential DDI.

In summary, the present study is the first to demonstrate that major cannabinoids and their metabolites inhibit morphine phase II metabolism mediated by UGT2B7. THC and CBD were shown to be the most potent inhibitors, exhibiting K_i,u_ values up to 3-fold lower than those observed for cannabinoid metabolites. Results from the present study show that the major route of metabolism for morphine (i.e., glucuronidation by UGT2B7) is strongly inhibited by numerous cannabinoids and their metabolites, suggesting that deleterious DDI may occur in individuals who concomitantly use cannabis or specific cannabinoids with morphine.

## Figures and Tables

**Table 1:** *IC_50_* and *IC_50,u_* values (µM) of THC, 11-OH-THC, 11-COOH-THC, CBD, and 7-OH-CBD for inhibition of UGT2B7-mediated morphine metabolism.

Metabolite	Enzyme ^a^	THC		11-OH-THC		11-COOH-THC		CBD		7-OH-CBD	
		*IC_50_*	*IC_50,u_* ^b^	*IC_50_*	*IC_50,u_* ^b^	*IC_50_*	*IC_50,u_* ^b^	*IC_50_*	*IC_50,u_* ^b^	*IC_50_*	*IC_50,u_* ^b^
Morphine-3-Glucuronide	rUGT2B7	20 ± 8.1	0.85 ± 0.34	12 ± 3.5	0.96 ± 0.27	47 ± 15	3.7 ± 1.2 ^c^	9.1 ± 4.3	0.35 ± 0.17	38 ± 27	3.0 ± 2.1 ^c^
	HLM	24 ± 5.5	1.2 ± 0.27	18 ± 17	1.7 ± 1.6	184 ± 37	17 ± 3.5 ^c^	8.2 ± 4.5	0.42 ± 0.23	17 ± 6.1	1.6 ± 0.57 ^c^
Morphine-6-Glucuronide	rUGT2B7	8.1 ± 1.6	0.34 ± 0.07	12 ± 3.6	0.96 ± 0.28	55 ± 15	4.3 ± 1.2 ^c^	4.9 ± 1.7	0.19 ± 0.06	39 ± 9.0	3.0 ± 0.70 ^c^
	HLM	15 ± 3.3	0.74 ± 0.16	13 ± 10	1.2 ± 0.95	104 ± 16	9.8 ± 1.5 ^c^	6.3 ± 2.6	0.32 ± 0.13	8.0 ± 2.5	0.75 ± 0.24 ^c^

^a^ r, recombinant enzyme from over-expressing cell microsomes; HLM, human liver microsomes. ^b^ *IC_50,u_* values are the mean (± S.D.) in µM and were corrected for non-specific binding with previous fraction unbound (*f_u,inc_*) values determined by Nasrin et al., 2021 [37,38]. ^c^ The *f_u,inc_* for 11-OH-THC was used as a surrogate value for calculating *IC_50,u_*.

**Table 2:** Calculated *K_i_* and *K_i,u_* (µM) values of THC, 11-OH-THC, 11-COOH-THC, CBD, and 7-OH-CBD for inhibition of UGT2B7-mediated morphine metabolism in HLM.

Inhibitor	Morphine-3-Glucuronide		Morphine-6-Glucuronide	
	Calculated *K_i_* ^a^ (µM)	Calculated *K_i,u_* ^b^ (µM)	Calculated *K_i_* ^a^ (µM)	Calculated *K_i,u_* ^b^ (µM)
THC	12.2 ± 2.77	0.59 ± 0.13	7.69 ± 1.63	0.37 ± 0.08
11-OH-THC	8.82 ± 8.42	0.82 ± 0.79	6.61 ± 5.02	0.62 ± 0.47
11-COOH-THC	91.6 ± 18.5	8.61 ± 1.74 ^c^	52.1 ± 8.16	4.89 ± 0.77 ^c^
CBD	4.11 ± 2.24	0.21 ± 0.11	3.16 ± 1.32	0.16 ± 0.07
7-OH-CBD	8.59 ± 3.05	0.81 ± 0.29 ^c^	3.99 ± 2.54	0.38 ± 0.12 ^c^

^a^ *K_i_* values were generated from *IC_50_* values, assuming competitive inhibition $\left(K_{i}=\frac{{IC}_{50}}{2}\right)$. ^b^ *K_i,u_* values were corrected with previous fraction unbound studies from Nasrin et al. 2021 [37,38]. ^c^ The *f_u,inc_* for 11-OH-THC was used as a surrogate for calculating *K_i,u_*.

**Table 3:** Prediction of clinical UGT-mediated cannabis—morphine drug interaction via mechanistic static modeling after inhaled and oral doses of THC and CBD.

Cannabinoid	Dose (mg) ^a^	Route of Administration	Morphine AUCR ^c^
THC	20	Oral	1.07
	130	Oral	**1.41 ^b^**
	160	Oral	**1.48**
	25	Inhalation	1.10
	70	Inhalation	**1.26**
	100	Inhalation	**1.35**
11-OH-THC	20	Oral	1.03
	130	Oral	1.18
	160	Oral	1.21
	25	Inhalation	1.04
	70	Inhalation	1.10
	100	Inhalation	1.14
11-COOH-THC	20	Oral	1.01
	130	Oral	1.03
	160	Oral	1.04
	25	Inhalation	1.01
	70	Inhalation	1.02
	100	Inhalation	1.02
CBD	70	Oral	**1.29**
	700	Oral	**2.36**
	2000	Oral	**2.87**
	19	Inhalation	1.10
7-OH-CBD	70	Oral	1.11
	700	Oral	**1.82**
	2000	Oral	**2.69**
	19	Inhalation	N.D. ^d^

^a^ Doses and C_max_ used to predict AUCR were reported from Bansal et al., 2022 [56]. The doses used for modeling 11-OH-THC and 11-COOH-THC were from administered doses of THC, while 7-OH-CBD was modeled using administered doses of CBD. ^b^ Bold values indicate AUCR values that are ≥ 1.25 as recommended by the FDA. ^c^ Morphine AUCR values were calculated utilizing *K_i,u_* from inhibition of morphine-3-glucuronide formation. ^d^ N.D., not determined.

## Data Availability

The original contributions presented in the study are included in the article/Supplementary Materials, further inquiries can be directed to the corresponding authors.

## Supplementary Materials

The following supporting information can be downloaded from: https://www.mdpi.com/article/10.3390/pharmaceutics16030418/s1, Figure S1: Representative IC_50_ curves in HLM against morphine-3-glucuronide and morphine-6-glucuronide formation; Table S1: Estimated maximum plasma concentrations of THC and CBD and their metabolites used to predict the magnitude of in vivo cannabinoid—drug interactions after consumption of cannabis by either oral or inhalation route.
