# Supplementary material for: Cannabinoid-Induced Inhibition of Morphine Glucuronidation and the Potential for In Vivo Drug–Drug Interactions
Source: Pharmaceutics. 2024 Mar 18;16(3):418. doi: 10.3390/pharmaceutics16030418 (PMC10975434; doi:10.3390/pharmaceutics16030418)
Supplement: Supplementary file 1 [file pharmaceutics-16-00418-s001.zip › pharmaceutics-2846648-supplementary.pdf]

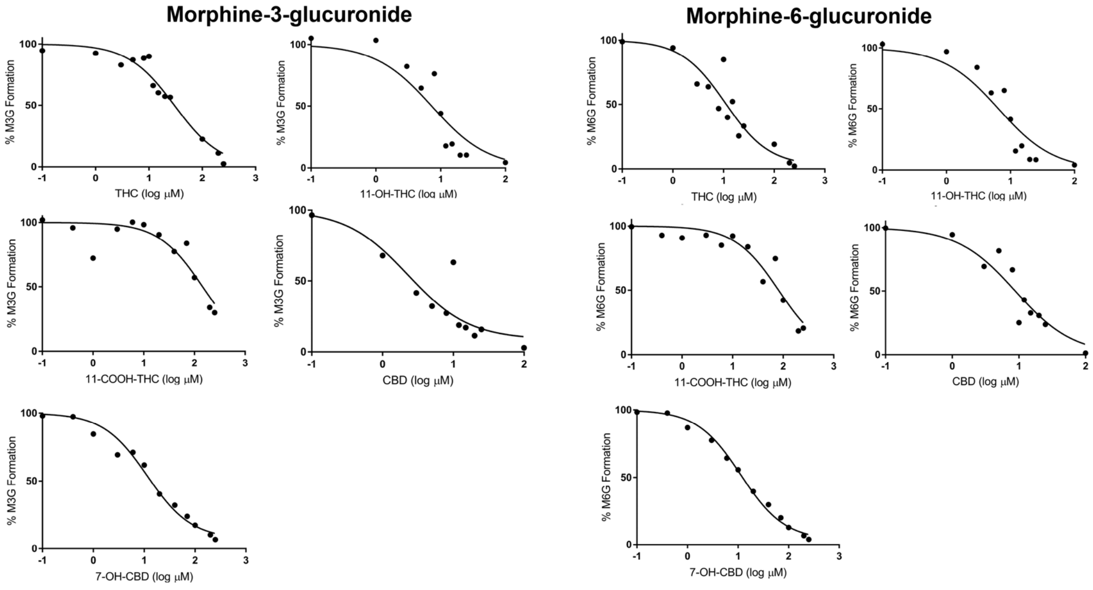

Figure S1: Representative IC_{50} curves in HLM against morphine-3-glucuronide and morphine-6-glucuronide formation

Table S1. Estimated maximum plasma concentrations of THC and CBD and their metabolites used to predict the magnitude of *in vivo* cannabinoid – drug interactions after consumption of cannabis by either oral or inhalation route.

Cannabinoid	Dose (mg)[a]	Route of Administration	$C_{max,hepatic\ inlet,u}$ (μM)
**THC**	20	Oral	0.03
	130	Oral	0.20
	160	Oral	0.24
	25	Inhalation	0.25
	70	Inhalation	0.69
	100	Inhalation	0.99
**11-OH-THC**	20	Oral	0.02
	130	Oral	0.10
	160	Oral	0.12
	25	Inhalation	0.01
	70	Inhalation	0.03
	100	Inhalation	0.05
**11-COOH-THC**	20	Oral	0.19
	130	Oral	1.22
	160	Oral	1.50
	25	Inhalation	0.07
	70	Inhalation	0.18
	100	Inhalation	0.26
**CBD**	70	Oral	0.09
	700	Oral	0.89
	2000	Oral	2.54
	19	Inhalation	0.35[c]
**7-OH-CBD**	70	Oral	0.03
	700	Oral	0.34

2000	Oral	0.98
19	Inhalation	N.D.[b]

[a] Doses and C_{max} used to predict AUCR were reported from Bansal et al., 2022 [56]. Doses used for modeling 11-OH-THC and 7-OH-CBD were from administered doses of THC and CBD, respectively. $C_{max,hepatic\ inlet,u} = f_{u,p} \times \left(C_{max} + \frac{F_a \times F_g \times K_a \times Dose}{Q_h \times R_b}\right)$, where $f_{u,p}$ (unbound fraction in plasma) was set to 0.03 (Garrett and Hunt, 1974) [52], F_a (fraction absorbed) and F_g (dose that escapes gut metabolism) for THC and CBD were set to 1 (FDA Drug Interactions Guidance, 2020) [53], k_a for both THC and CBD was set to 0.02 (Cox et al., 2019) [45], Q_H (hepatic blood flow) – 1500 mL/min, and BP (blood to plasma ratio) for THC and CBD was set to 0.4 (Schwilke et al., 2009) [55]. [b] N.D., not determined. [c] Doses and C_{max} used to predict AUCR were reported from Cox et al., 2019 [45].
